# Anastomosing Haemangioma: Report of Three Cases With Molecular and Immunohistochemical Studies and Comparison With Well-Differentiated Angiosarcoma

**DOI:** 10.3389/pore.2022.1610498

**Published:** 2022-08-01

**Authors:** Yi-Che Chang Chien, Livia Beke, Gábor Méhes, Attila Mokánszki

**Affiliations:** Department of Pathology, Faculty of Medicine, University of Debrecen, Debrecen, Hungary

**Keywords:** angiosarcoma, anastomosing haemangioma, immunohistochemistry (IHC), fluorescence *in situ* hybridization (FISH), next-generation sequencing (NGS), GNA11 mutation

## Abstract

Anastomosing haemangioma (AH) is a newly described distinct vascular neoplasm that histologically may confuse with well-differentiated angiosarcoma (AS) for those who are unfamiliar with this rare entity. We aimed to identify molecular genetic differences between AHs and ASs by carrying out immunohistochemistry (IHC), fluorescence *in situ* hybridization (FISH), and next-generation sequencing (NGS). Immunohistochemically, all six cases showed positivity for cyclinD1 and pERK. All cases of AH showed focal weak positive reaction for p53 and MIB-1, and the IHCs for HIF-1α were all negative for all three cases. Those three cases of angiosarcoma revealed strong, diffuse positivity for p53, 50%–70% MIB-1 labelling, and multifocal, moderate to strong HIF-1α expression. To further clarify the difference in p53 expression, we carried out a FISH which revealed 17p polysomy in all three ASs whereas copy number aberration was absent in the AH group. In one AH case, the *GNA11* c.627G > T nucleotide variant was detected. Due to the rarity and overlapping morphological features, AH might be difficult to separate from other vascular tumours, in particular from well-differentiated AS also featured by mild hyperchromatic, hobnail-like endothelial cells. The potential molecular differences between these two entities presented here may be used in support of the correct diagnosis.

## Introduction

Anastomosing haemangioma (AH) is a newly described distinct vascular neoplasm first reported in the genitourinary system. Later, distinct anatomical locations have also been reported including in the visceral organs [1–3] and paraspinal soft tissue [3]. Morphologically, AHs are characterized by loosely lobulated proliferations of capillary-like vessels with anastomosing, sinusoid patterns. They are lined by endothelial cells exhibiting hobnail-like appearances with mildly hyperchromatic nuclei protruding into the lumen, which is often confused with well-differentiated angiosarcoma (AS) for those who are unfamiliar with this rare entity. On the other hand, AHs also contain intravascular thrombi, intracytoplasmic eosinophilic hyaline globules and occasionally, extramedullary haematopoiesis, whereas multilayering nuclei, brisk mitotic activity and infiltrating into the adjacent tissue are usually absent. Recently, it has been reported that approximately 70% of AHs harbour activating *GNAQ* mutations, specifically at codon 209, providing molecular evidence of the neoplastic nature of AHs [4]. Furthermore, activating mutation *GNA11* (codon 209), and *GNA14* (codon 205), the paralogues of *GNAQ* have also been documented indicating the molecular heterogeneity of AHs [5, 6]. Mutations of *GNAQ*, *GNA11* and *GNA14* further trigger the MAPK signalling pathway which leads to uveal melanoma and congenital haemangioma formations. This article will investigate the potential molecular genetic differences between AHs and ASs by carrying out immunohistochemistry (IHC), fluorescence *in situ* hybridization (FISH), and next-generation sequencing (NGS) molecular analysis from three AHs and three ASs.

## Materials and Method

### Histology and Immunohistochemistry

Haematoxylin and eosin (HE)-stained sections of all cases were reviewed by the same pathologist. After sectioning of 4 μm slides from formalin-fixed, paraffin-embedded blocks, deparaffinization in xylene and rehydration in a series of decreasing concentrations of ethanol were performed. Antigen retrieval using either the Bond Epitope Retrieval Solution 1 (pH∼6) or the Bond Epitope Retrieval Solution 2 (pH∼9) (Leica Microsystems, Wetzlar, Germany) was carried out at 99∼100°C for 20∼30 min. The slides were then treated with cyclinD1 (ready to use, clone SP4-R, Roche, Basel, Switzerland), p53 (1:700, clone Do-7, Dako, Agilent Technologies Company, Santa Clara, CA, United States), pERK (1:200, clone D13.14.4E, Cell Signaling Technology, Danvers, MA, United States), HIF-1α (1:400, polyclonal, GeneTex, Irvine, CA, United States) and MIB-1 (1:100, clone MIB-1, Dako, Agilent Technologies Company, Santa Clara, CA, United States) separately. Immunostaining was conducted on the Leica BOND-MAX™ autostainer (Leica Microsystems, Wetzlar, Germany) and the peroxidase/DAB Bond™ Polymer Refine Detection System (Leica Microsystems, Wetzlar, Germany) was utilized for visualization purposes.

All protocols have been approved by the author’s respective Institutional Review Board for human subjects (IRB reference number: IV/8465-3/2021/EKU).

### Fluorescence *In Situ* Hybridization

Fluorescence *in situ* hybridization (FISH) was performed using XL TP53/17cen dual colour deletion probe to detect *TP53* gene amplification/deletion on FFPE samples according to the manufacturer’s protocol (Metasystems, Altlussheim, Germany). The probe contains the chromosome 17 centromere for ploidy control. Denaturation and hybridization were performed in a hybridization chamber (StatSpin ThermoBrite, Abbott Laboratories, Abbott Park, IL, United States). Slides were denatured at 85°C for 5 min, and hybridization was subsequently performed overnight at 37°C. Fluorescence images were archived with the Isis imaging system (MetaSystems, Altlussheim, Germany).

### Next-Generation Sequencing

QIAamp DNA FFPE Tissue Kit (Qiagen, Hilden, Germany) was used for FFPE tissue DNA extraction. The isolations were carried out according to the manufacturer’s instructions. The DNA concentrations were measured in the Qubit dsDNA HS Assay Kit using a Qubit 4.0 Fluorometer (Thermo Fisher Scientific, Waltham, MA, United States). For NGS analysis, libraries were constructed using AmpliSeq for Illumina Focus Panel (Illumina, San Diego, CA, United States) targeted resequencing assay for 52 genes with known relevance to solid tumours, and Accel-Amplicon Comprehensive *TP53* Panel (Swift Biosciences, Integrated DNA Technologies, Coralville, IA, United States) for *TP53* sequencing.

The indexed libraries were then presented to Illumina MiSeq System (MiSeq Reagent kit v3 600 cycles, Illumina, San Diego, CA, United States). The libraries pooling, denaturation and dilution were carried out according to the manufacturer’s protocol. The finished loading concentration was 8 pM libraries and 1% PhiX. Captured libraries were sequenced with a paired-end run to obtain 2 × 150 bp reads with at least 250X depth of coverage.

The fastq files were analyzed with the NextGENe software (version 2.4.2; SoftGenetics, State College, PA, United States) for identifying single-nucleotide variants (SNVs) as well as insertions and deletions (indels). For the alignment, the human reference genome GRCh37 (equivalent UCSC version hg19) was used. The sequence quality was evaluated and the cutoff was determined to be 5% variant allele frequency (VAF). Massive insertion/deletion (>50 bp) and compound structural changes can not be captured by this method. The results were determined using the latest version of the Human Genome Variation Society nomenclature.

## Results

### Clinicopathological Features

The six cases of AHs and ASs included in this study were diagnosed at our institute between 2018 and 2021. For the AH group, all three patients were female with an age bracket of 52–76 years old. Two cases presented with AH within the ovary, and one from the kidney. All three cases were well-circumscribed, and the size varied between 1.8 and 3.5 cm. All three cases received complete surgical excision. For the AS group, one male and two female patients were included with ages between 44 and 81 years old. The tumours arose from the chest wall, breast, and brain, respectively and the size ranged between 2.3 and 3.6 cm with irregular borders and incomplete excision. Further clinical information is illustrated in Table 1.

**TABLE 1:** Clinicopathological information of anastomosing haemangioma and angiosarcoma cases (DOD, died of disease. NET, neuroendocrine tumour).

Diagnosis	ID	Gender	Age	Location	Size (cm)	Margin	Complete excision	MIB-1 (%)	CyclinD1	pERK	p53	CA-IX	HIF-1α	NGS Result	Follow-up
Anastomosing Haemangioma	1	F	68	Right ovary	1.8	Well circumscribed	Yes	1	Strong, diffuse	+	Weak, focal	—	—	Negative	Alive
	2	F	76	Right ovary	3.5	Well circumscribed	Yes	1	Strong, diffuse	+	Negative	—	—	Negative	DOD (NET of the duodenum)
	3	F	52	Right kidney	1.2	Well circumscribed	Yes	1	Strong, diffuse	+	Weak, focal	—	—	*GNA11* positive	Alive
Well-differentiated Angiosarcoma	4	F	68	Right chest wall	2.7	Poorly circumscribed	No	30	Strong, diffuse	+	Strong, diffuse	—	N/C+	Negative	DOD after 9 months
	5	M	44	Brain	3.6	Poorly circumscribed	No	20	Strong, diffuse	+	Moderate, diffuse	—	N+	Negative	DOD after 4.5 months
	6	F	81	Breast	2.3	Poorly circumscribed	No	45	Strong, diffuse	+	Moderate, diffuse	—	N+	*MAP2K1* and *MYC* positive	DOD after 13 months

A good prognosis was recorded for the AH group, as two out of the three patients remain alive (based on follow-up data). The third patient, however, passed away due to complications from another tumour (grade 1 neuroendocrine tumour of the duodenum). A poor prognosis is seen in the AS group as all three cases died of disease within the follow-up period of 4.5 and 13 months after the initial pathological diagnosis.

### Histological Features Including Immunohistochemistry

**FIGURE 1:**
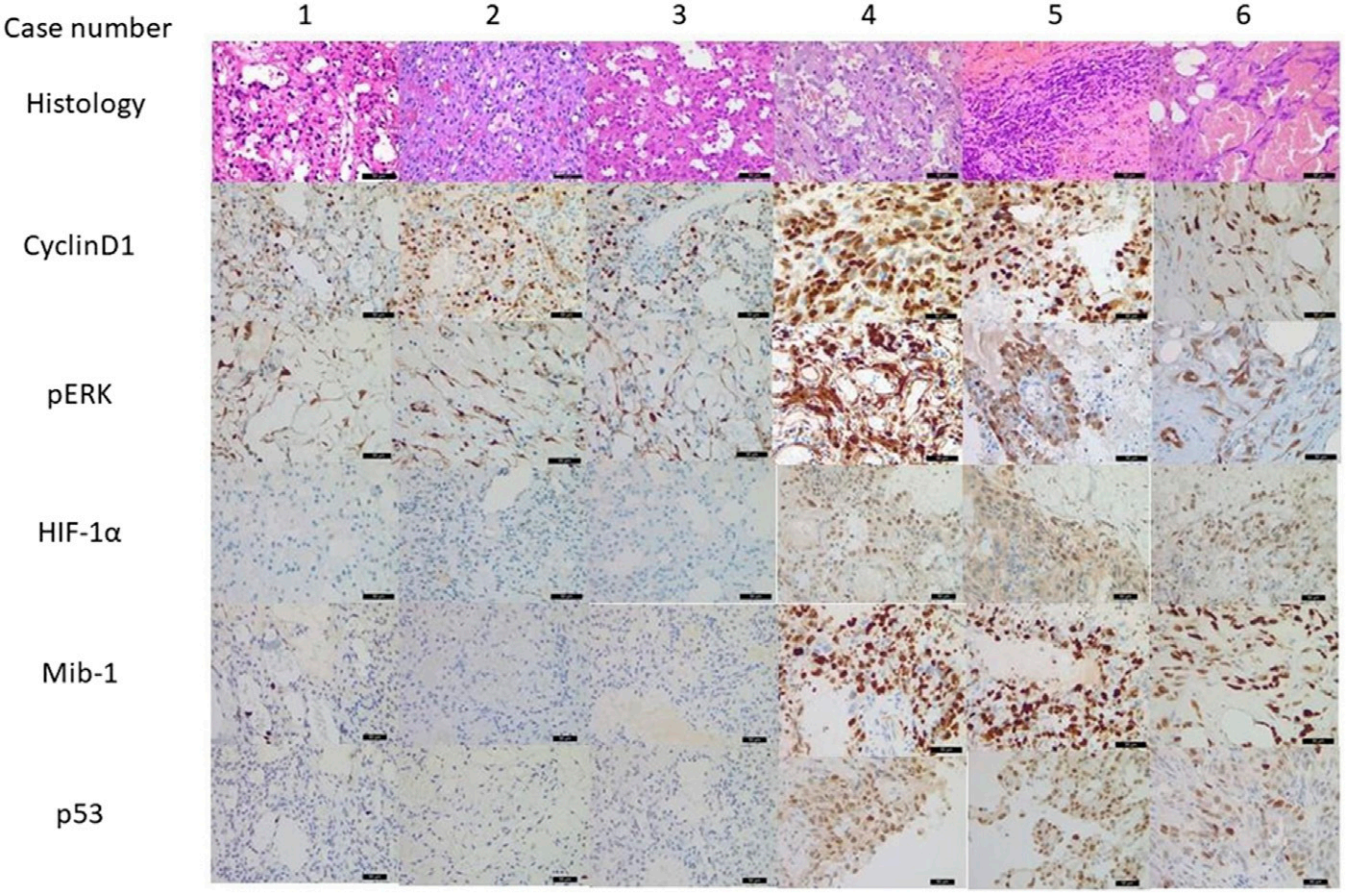
Morphology and immunohistochemistry result from anastomosing haemangiomas (AH, case 1–3) and angiosarcomas (AS, case 4–6) (×400 magnification). All six cases showed positivity for cyclinD1 and pERK indicating MAPK pathway activation. AHs also showed focal weakly positive for p53 and low MIB-1 positivity, and HIF-1α were negative for all three cases. ASs revealed strong, diffuse positivity for p53, significant higher MIB-1 labelling, and multifocal, moderate to strong HIF-1α expression.

The histologic features of all AHs consisted of capillary-sized vascular channels in lobulated and solid patterns with rare mitoses. Hobnailing of endothelial cells with mild cytologic atypia was also noted (Figure 1 case 1–3). Vessels containing thrombi and intracytoplasmic eosinophilic globules were found in all three cases, whereas extramedullary haematopoiesis was absent. The stroma was fibrosclerotic with focal myxoid degeneration. Red blood cell extravasation, hemosiderin pigment and intertumoral adipose tissue can be found as well. The peripheral areas show chronic inflammatory cell infiltration. No noticeable necrosis was seen. The three angiosarcoma cases (Figure 1 case 4–6), on the contrary, showed bizarre nuclear atypia, infiltrative growth pattern with significant mitosis and tumour necrosis. Immunohistochemically, all six cases showed positivity for cyclinD1 and pERK. All cases of AH showed focal weakly positive for p53 and MIB-1 labelling index (less than 1%), and the IHCs for HIF-1α were all negative for all three cases. Those three cases of angiosarcoma revealed strong, diffuse positivity for p53, 50%–70% for MIB-1, and multifocal, moderate to strong HIF-1α expression (two cases intranuclear and one case intranuclear and cytoplasmic positivity).

### Fluorescence *In Situ* Hybridization

**FIGURE 2:**
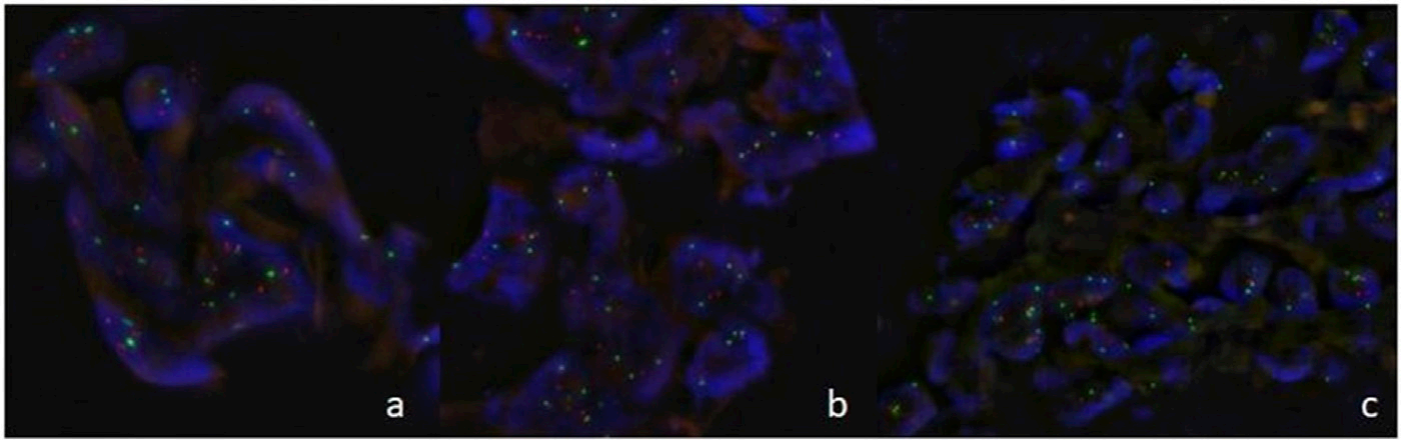
Fluorescence *in situ* hybridization in angiosarcomas (**(A)**: case 4, **(B)**: case 5, **(C)**: case 6) showed of multiple dots for *TP53* gene (orange) and chromosome 17 centromere (green) indicating *TP53* gene polysomy.

To further clarify the difference in p53 expression between AHs and ASs, we carried out a FISH examination which revealed polysomy in all three ASs (Figure 2). Genetic aberration was absent in the AH group.

### NGS-Based Mutation Analysis

Using the AmpliSeq Focus Panel in one AH case the *GNA11* c.627G > T; p. Q209H nucleotide variant was detected with 11% VAF. The other two AH cases were considered negative concerning this NGS panel.

When sequencing the AS samples, the molecular aberration was detected in only one case. *MAP2K1* c.166C > A; p.Q56K and *MYC* c.217A > C; p.T73P alterations were observed in the positive AS sample with 37.97% and 22.3% VAF, respectively.

Because of differences between AH and AS cases p53 IHC staining, NGS was performed, which did not detect SNVs and small indels in the *TP53* gene.

## Discussion

**FIGURE 3:**
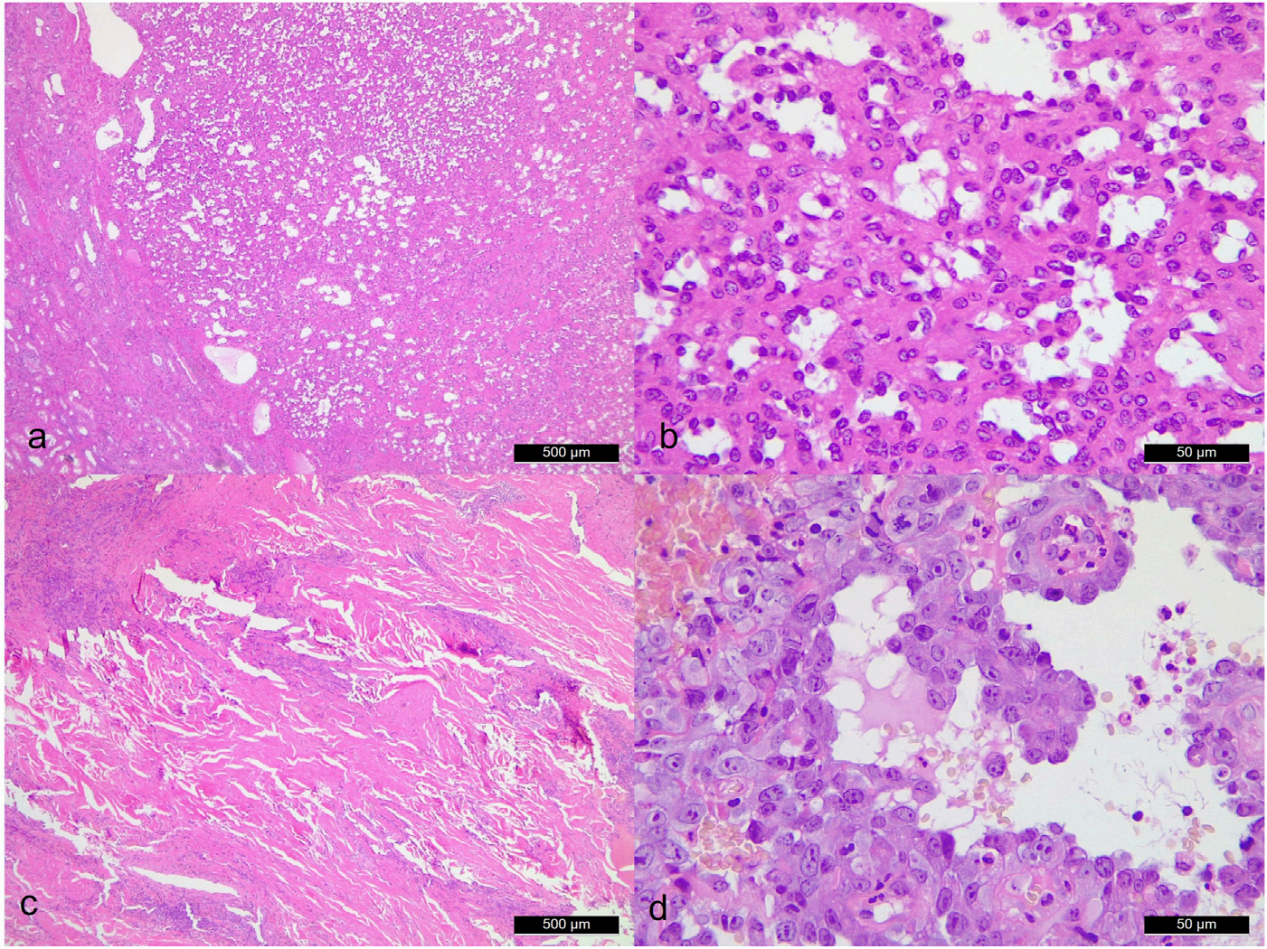
Morphological comparison between anastomosing hemangioma [case 3. **(A)**: ×40 magnification. **(B)**: ×400 magnification] and angiosarcoma [case 4. **(C)**: ×40 magnification. **(D)**: ×400 magnification]. Angiosarcoma shows significant cytologic atypia, endothelial cell multilayering and broadly infiltrative pattern.

Due to its rarity and overlapping morphological features, AH may be confused with other vascular tumours particularly well-differentiated AS due to the mild hyperchromatic, hobnail-like endothelial cells. Nevertheless, AHs usually possess differentiating factors such as the presence of lobulated contours and absence of prominent nuclear atypia, multilayering nuclei, high mitotic activity, infiltrating-dissecting growth pattern and massive tumour necrosis (Figure 3). In our study, the mitotic activity labelled by MIB-1 in our three AH cases was less than 1% compared ASs (>50%). Additionally, intravascular fibrin thrombi, peripheral adipose tissue, and, although not in our cases, extramedullary haematopoiesis can also serve as additional diagnostic clues for AHs. Furthermore, radiological information is crucial to separate AH from angiosarcoma as well [7].

*GNAQ* encodes G-protein subunit alpha q, together with its paralogues, *GNA11*, *GNA14,* and *GNA15*, comprise the alpha q subfamily of G proteins, which linked G-protein coupled receptors are the upstream components of ERK. It is known that mutations in *GNAQ* at codon 209, alter a region within the catalytic GTPase domain, resulting in constitutive activity which contributes to the oncogenesis of blue naevus and uveal melanoma [8]. Mutual exclusivity among *GNAQ* and its paralogue mutations have also been documented [9]. In our study, we identified one case of an AH harboured *GNA11* mutation at codon 209, while the other two failed to present with hotspot mutations at neither *GNAQ* nor *GNA11* ***.*** Due to the restriction of our molecular study panel, which only assayed *GNAQ* and *GNA11*, we cannot exclude the possibility that two cases lacking mutations in *GNAQ*, and *GNA11* might carry a mutation in the other genes within the signalling pathway. Intriguingly, a *GNAQ/11* mutation is not entirely specific for AH as other types of vascular lesions such as congenital vascular malformation or cherry haemangioma [6, 10, 11] also carry the same genetic mutation. This implies the significance of clinical and morphological correlation to achieve the correct diagnosis.

The mitogen-activated protein kinase (MAPK) cascade is a highly conserved module involved in various cellular functions including cell proliferation, differentiation, and migration. Several proteins are involved in this pathway including extracellular signal-regulated kinases (ERK), which connect to G proteins through a multitude of distinct signal transduction pathways under dual phosphorylation on Tyr204 and Thr202 for ERK1 (p44), and Tyr187 and Thr185 for ERK2 (p42). Once activated, a signal is transmitted by phosphorylation of neighbouring proteins [12, 13]. The highly sensitive monoclonal antibody used in our study directly targets ERK1 (pThr202/pTyr204) and ERK2 (pThr185/pTyr187) and can serve as direct evidence of MAPK activation. In our study both AHs and ASs show both intranuclear and intracytoplasmic staining, together with intranuclear cyclinD1 positivity, implying the MAPK pathway is activated in both tumour groups, which is not surprising since it is crucial for angiogenesis [14]. However, we found the AS group revealed strong p53 expression, to further clarify this result, we carried out FISH which revealed *p53* polysomy in all three ASs and a negative p53 result in the AH cases. Furthermore, we are interested in whether the microenvironment might play a role in these two entities by staining HIF-1α which shows two cases of multifocal intranuclear and one case of both intracytoplasmic and intranuclear positivity in angiosarcoma and negative for all AHs. Our data indicate that the MAPK pathway is triggered in both AH and AS by different genetic aberrations.

Apart from angiosarcomas, AH should also be differentiated from other vascular neoplasms as well such as retiform hemangioendothelioma, hobnail hemangioma, and sinusoidal hemangioma. They typically involve the skin and deep soft tissue or visceral occurrences are uncommon. Additionally, retiform hemangioendotheliomas show long, branching vascular channels lined by distinctly hyperchromatic, hobnailed endothelial cells with intraluminal papillations. Hobnail hemangiomas often have a distinctive clinical appearance and grow in a biphasic, wedge-shaped pattern, with dilated, superficial vessels lined by hobnail endothelial cells and a deeper dermal proliferation of capillaries forming slitlike spaces. Sinusoidal hemangiomas posses larger, gaping vessels with fibrotic walls and tend to lack adipocytic metaplasia, small thrombi, and extramedullary hematopoiesis, which is relatively common in AHs.

In conclusion, in our manuscript, we aimed to characterize general pathological features of AHs and compare them with a small selection of AS cases. While the three AH samples from the same institution represent a significant collection due to the rarity of the lesion, relatively more frequent ASs was included to highlight basic morphological and biological differences reflected by our setting. Regarding the specific molecular characteristics in AS our results were in close agreement with earlier publications, demonstrating the involvement of the *TP53* gene [15]. We also demonstrated one case of AHs harbouring a *GNA11* mutation which, besides conforming to its clonal nature, also serves as an important molecular signature to distinguish AHs from well-differentiated ASs. We addressed the potential molecular differences between these two entities. Nevertheless, due to the small sample size and its rarity, a larger scale study is needed to elucidate this issue. Clinical information and histology feature still serve as the gold standard for the correct diagnosis.

## Data Availability

The data presented in this study is available upon request from the corresponding author. The data is not publicly available due to the protective rights of the patients.
